# State-Anxiety and Academic Burnout Regarding University Access Selective Examinations in Spain During and After the COVID-19 Lockdown

**DOI:** 10.3389/fpsyg.2021.621863

**Published:** 2021-01-27

**Authors:** Antonio Fernández-Castillo

**Affiliations:** Department of Developmental and Educational Psychology, University of Granada, Granada, Spain

**Keywords:** anxiety, test-anxiety, burnout, university access, evaluation, educational psychology, health emergency, COVID-19

## Abstract

Coping with assessment tests are known to generate anxiety frequently in the students who face them. In academic circumstances with the continued presence of emotional disturbance, high demand, and stress, emotional and physical fatigue, typical of burnout syndrome, and can be detected. Anxiety and burnout are related to each other and even more closely in high-stakes tests. One of these tests is the examination imposed in Spain for access to the university. The objective of this work is to analyze the presence of anxiety and burnout and the relationship between them in students who face these tests, both during the confinement situation due to the COVID-19 pandemic and during the pandemic after the lockdown. For this purpose, we used a sample of 1,021 students with a mean age of 17.89 (SD = 1.22, range 17–27). Of these, 866 (84.8%) were students who were taking the test, while the rest were university students who had passed it recently. Our results show high levels of anxiety and burnout in students who face the evaluation test during the COVID-19 pandemic, sustained over time and especially in comparison with students who had already taken the exam. The association between higher levels of anxiety and higher levels of burnout in the students who take these exams was also verified. These results link the relationship between these two variables more solidly and suggest the need to include address anxiety to reduce burnout levels in these students. The results are discussed with regard to prior evidence and their applications.

## Introduction

Given the escalation in the number of COVID-19 infections at the beginning of March 2020 in Spain, the government of the nation, after the convocation of an extraordinary council, declared a state of alarm on March 14 of that year (Royal Decree 462/2020., 2020). This declaration came just 3 days after the World Health Organization raised the public health emergency caused by COVID-19 to an international pandemic on March 11, 2020 (World Health Organization, 2020).

The main decision derived from that declaration by the Spanish government was the imposition of a national quarantine. The measure came into force at 00:00 h on Sunday, March 15, and implied forced isolation of all Spanish citizens and foreigners residing in Spain (Jones, 2020; Royal Decree 462/2020., 2020).

On April 28, the government announced the so-called the “Deconfinement Plan,” consisting of four phases that concluded in the “new normality” on June 21, thus ending the state of alarm at 00: 00 h of that day (Sánchez and Álvarez, 2020). During that period of the state of alarm (and afterward, until September 2020), the educational system ceased working in person, teaching exclusively online. This operation affected various areas, with final-year high school students being particularly affected, especially because preparation for the selective university entrance exams takes place during this course. This year, the tests for the June call were delayed to ensure better health security conditions and were carried out, in person, in the first half of July (Yaq.es., 2020). Preparation for the tests in these circumstances was clearly not the same as that of previous courses, depending fundamentally on the students’ individual work at home and in isolation. It should be clarified that in Spain, as in other countries, university entrance exams are the access route to public universities. Likewise, the choice of career depends almost directly on the note obtained in them.

Some studies have already shown how the confinement situation in different countries has been associated with anxiety, mood changes, changes in daily life patterns (including increased consumption of tobacco and alcohol, longer exposure to screens, etc.) and worsening health-related behaviors, among other variables (Alemany-Arrebola et al., 2020; López-Bueno et al., 2020a,b; Smith et al., 2020).

Getting tired of performing activities continuously is normal. When these activities are inherent to work or responsibility and are carried out for long periods of time (i.e., years), they can lead to chronic fatigue, popularly referred to as being “burnt.” In Psychology, for a long time, this state has been called burnout and has been conceptualized as a state of emotional, physical, and cognitive exhaustion as a consequence of performing responsible activities under demand and stress (Barraza, 2011). Burnout is classically characterized by three sets of manifestations: (a) fatigue, which is the main manifestation; (b) depersonalization and distancing from the required performance, with manifestations such as low achievement and abandonment; and (c) loss of commitment and decrease in personal achievement and motivation (Maslach and Jackson, 1986; Rodríguez-García et al., 2017). Although at first, burnout was detected and studied in work and professional contexts, its study was soon extended to the educational context. In this area, it was observed that burnout could be associated with lower levels of academic achievement and performance, and low self-efficacy and motivation, among other aspects (Rahmati, 2015; Fiorilli et al., 2017). In students, stress and overload over an extended time have been considered etiological factors that explain the presence of exhaustion and lack of motivation (Yang, 2004), and, like in professional performance, absenteeism, dropout and low satisfaction with studies (Rahmati, 2015; Fiorilli et al., 2017; Salmela-Aro and Read, 2017). In the educational context, one of the circumstances that generates more demand and continued stress in the students is the exams. Coping with exams has also been associated with students’ burnout and emotional exhaustion (Yildirim, 2007; Fernández-Castillo et al., 2020), anxiety (Fernández-Castillo et al., 2009) and, possibly, great concern.

Worrying about things of daily life is normal. However, worrying excessively and continuously or without justified cause is one of the most commonly recognized symptoms of anxiety or any of its associated disorders (American Psychiatric Association., 2014; National Institute of Mental Health., 2020). Anxiety as a personality trait and anxiety as a reaction to situations that could generate it have been differentiated for a long time (Spielberger et al., 2002). Anxiety disorders are among the most frequent worldwide, affecting more than 260 million people (World Health Organization, 2017).

In the educational context, anxiety is usually present at different times and situations, with evaluation tests being one of the most frequently associated situations (Fernandez-Castillo and Caurcel, 2019). In fact, according to some authors, exam anxiety is the most common type of anxiety in the educational setting (Brodersen, 2017; von der Embse et al., 2018).

Concerning exams, anxiety is understood as a state and is characterized by a feeling of fear and tension, worry, and a perception of threat, together with negative physiological manifestations (American Psychiatric Association., 2013), moreover, with some particular characteristics such as the fear of being negatively evaluated and of failing (Fernández-Castillo, 2013). It is a transitory state, activated by anticipation or exposure to an exam, which involves the described emotional disturbance (Spielberger et al., 2002).

Exam anxiety has been associated with other psychological disorders and problems, including generalized anxiety, attention deficit hyperactivity disorder, phobias, or depression, among others (Kavakci et al., 2011; Leadbeater et al., 2012; Jacobson and Newman, 2017).

Regarding academic functioning, exam anxiety has been related to many different negative aspects that can affect the result of the exam itself or performance in general (Fernández-Castillo and Gutiérrez-Rojas, 2009; von der Embse and Witmer, 2014; Dominguez-Lara et al., 2017), as well as increased risk of academic dropout (Esch et al., 2014) or difficulties in cognitive processes such as concentration and selective attention during the examination processes themselves (Fernández-Castillo and Caurcel, 2015). Some studies have also related anxiety to the presence of burnout in students (Chust-Hernández et al., 2019).

One aspect that seems to increase exam anxiety is its possible consequences. As these are perceived by students as very important or threatening, anxiety levels may be higher, with high-stakes tests being especially anxiogenic (Segool et al., 2013). The university access exam is one of the most important tests for students in many countries. This test not only allows access to higher education, but also career choice, and even professional performance in one’s life. Consequently, very high levels of anxiety have been detected during these tests in various countries (Yildirim, 2007; Kavakci et al., 2014; Unalan et al., 2017). In Spain, as in other countries, coping with these tests has been associated with burnout, poor performance, and emotional fatigue (Yildirim, 2007; Johnston et al., 2016; Fernández-Castillo et al., 2020). However, to date, we lack sufficient information about these circumstances.

Therefore, although the physical and emotional fatigue inherent to burnout and its association with anxiety during exams have been previously studied, not many studies have studied these aspects in subjects who face the university access exam in Spain. Still less attention was paid to the extraordinary situation of confinement suffered by these students between March and June 2020, when they were not attending the educational centers in-person to prepare this test.

Consequently, in this work, we propose the following objectives. Firstly, we analyzed the levels of anxiety and burnout in the participants. Secondly, we explored the association between anxiety and burnout in students who take the university access exam after having been recently confined.

Our third objective focuses on the evolution of anxiety and burnout sustained over time, as well as the levels in confined subjects compared with a control group. Finally, our fourth objective was to analyze possible differences in burnout according to different levels of anxiety in the students who take the access exam in a post-lockdown situation.

The reviewed previous evidence makes us hypothesize that there will be a significant association between anxiety and burnout in the students who face the test. Moreover, higher levels of burnout will be found in subjects who have higher levels of anxiety before the selectivity test. On the other hand, we expect to find that the levels of anxiety and burnout will be maintained over time, and that the levels will be higher in subjects confined by COVID-19 and those who were taking the test, compared to control subjects.

## Materials and Methods

In this research, the design used was a retrospective or ex post facto study (Sharma, 2019) with a cross-sectional design to collect data.

### Sample

In this research, participants were 1,021 students with a mean age of 17.89 years (Mode = 18, SD = 1.22, range 17–27). Concerning gender, 638 were female (62.5%), 368 were male (36%), four had chosen the option “others” (0.4%), and 11 (1.1%) did not provide this information. All of them studied either in the city of Granada or in its province, in Spain.

Of them, 866 (84.8%) were enrolled in their last year of high school: 390 (38.2%) were currently (during the assessment days) taking the university access exam, and 476 (46.6%) participants had been in a situation of national confinement due to the COVID-19 pandemic at least 2 month before taking it.

The remaining students (*n* = 155, 15.2%) were considered a control group, with assumed normal functionality (no alteration associated with the university access exam or the pandemic) because they had already taken the exam at least 3 month before the COVID-19 pandemic and were currently first-year university students. Of these, 60 studied Early Childhood Education, and 95 studied Sociology. These three groups (before taking the exam, during the exam, and already enrolled in the career (control group) formed the three groups of subjects according to the time/situation of data collection. Table 1 shows more descriptive data on the sample of participants.

**TABLE 1:** Descriptive data on the sample of participants.

		**Lockdown**	**Post-lockdown**	**Pre-COVID 19 (social normality)**	**Total**
Age [mean (SD)]		17.49 (0.63)	18 (1.16)	18.86 (1.88)	
Gender [*n* (%)]	Male	167 (35.1%)	145 (37.2%)	56 (36.1%)	368
	Female	308 (64.7%)	237 (60.8%)	93 (60%)	638
	Others		2 (0.5%)	2 (1.3%)	4
	Lost	1 (0.2%)	6 (1.5%)	4 (2.6%)	11
Total		476	390	155	1021

### Procedure

The data collection involved two procedures in three phases, which gave rise to the three groups of participants, making up the general sample.

The first and second data collection was carried out through an online survey (google forms) whose link was distributed to a sample of last-course high school students. For this purpose, we previously contacted the board of directors of various educational centers in the province of Granada (Spain) to request authorization and collaboration. Through them, a pool of specific professors was contacted who collaborated by distributing the request for participation and the link to the questionnaire among the students.

The request to the students was accompanied by specific information detailed below, as well as the request to sign an informed consent. Students who were preparing their access to the university responded to the survey between April 1 and 30, 2020, while those who were taking the university access exam did so between July 7 and 9 of that year (the date when the tests are held in Granada). In these cases, the survey could be completed through mobile devices or PCs.

The third set of data had been collected before the COVID-19 pandemic (between December 1 and 20, 2018) from first-year university students of the specialties of Sociology and Early Childhood Education in the faculties of Sociology and Education Sciences of the University of Granada. Authorization had been requested from the professors in whose classrooms the evaluation was carried out and the data were collected in groups.

The students who were evaluated during the university access exams were asked to fill in the questionnaire either at the end of the session of any of the tests that make up the exam or during the break in any of them, but not at the end when the exam had ended. In any case, the questionnaires were completed between July 7 and 9, 2020, either on the street or at home.

Before requesting their consent, participants were informed of the main objectives of the research, the anonymity of the information collected, and the voluntariness of their participation. They were also requested not to leave any item unanswered and to be sincere. The criteria established by the authors of each specific inventory were taken into account.

In this research, no personal data was collected that could allow the identification of the participants. The answers given in the questionnaires were transferred to a database for subsequent statistical analysis. Regulation (EU) 2016/679 of the European Parliament and of the Council of April 27, 2016 on the protection of natural persons with regard to the processing of personal data was followed at all times.

### Instruments

The evaluation instruments were assembled in a questionnaire headed by the request to report their age and gender. Concerning gender, the participant could choose “male,” “female,” or “other.”

The Spielberger State-Trait Anxiety Inventory (STAI), in its Spanish version (Spielberger et al., 2002), was used to assess participants’ anxiety. This is one of the most valid and reliable instruments for the assessment of anxiety (Spielberger et al., 1983; Fonseca-Pedrero et al., 2012), and provides an indicator of Trait-Anxiety or State-Anxiety The State-Anxiety subscale consists of 20 items that are rated on a four-point Likert scale ranging from 0 (not at all) to 3 (very much), providing a global score ranging between 0 and 60. It also presents a scale that establishes three groups according to the general score achieved (Spielberger et al., 2002): scores above 31 are considered a high level of anxiety, values between 15 and 30 express a medium level of anxiety, and scores below 14 indicate that the level of anxiety is low. Its psychometric properties are excellent, as confirmed, for example, by the results obtained in the Kuder–Richardson-20 test (KR-20), with values between 0.90 and 0.93, or the split-half reliability test, where the State-Anxiety subscale, which is the one used in the present study, reached a value of 0.94 (Spielberger et al., 2002). In our case, this subscale also reached excellent levels of internal consistency (Cronbach alpha): α = 0.92.

To quantify burnout in the participants, we used the one-dimensional Student Burnout Scale (Barraza, 2008, 2011) in its version adapted to the Spanish population (Fernández-Castillo et al., 2020). This scale provides a one-dimensional evaluation of academic burnout, where higher scores imply greater cognitive, emotional, and physical exhaustion in general or specific academic activities. This instrument presents 15 items that are rated on a four-point response scale ranging from 1 (never) to 4 (always), with a range of possible global scores between 15 and 60. Regarding its psychometric properties, using the split-half test (according to the Spearman-Brown formula for tests of Unequal-Length), the original studies found a reliability of 0.89. In the same study (Barraza, 2011), Cronbach’s alpha reached an excellent value (α = 0.91). In our sample, the value of Cronbach’s alpha was α = 0.87.

### Data Analysis

Data analysis included descriptive and frequency analysis, correlation analysis (specifically, a Pearson bivariate correlation test), and means comparisons tests, specifically one-way ANOVA, with analysis of minimally significant difference in some cases. The analyses were carried out with the SPSS statistical software, assuming in all tests a level of significance less than 0.05.

## Results

**FIGURE 1:**
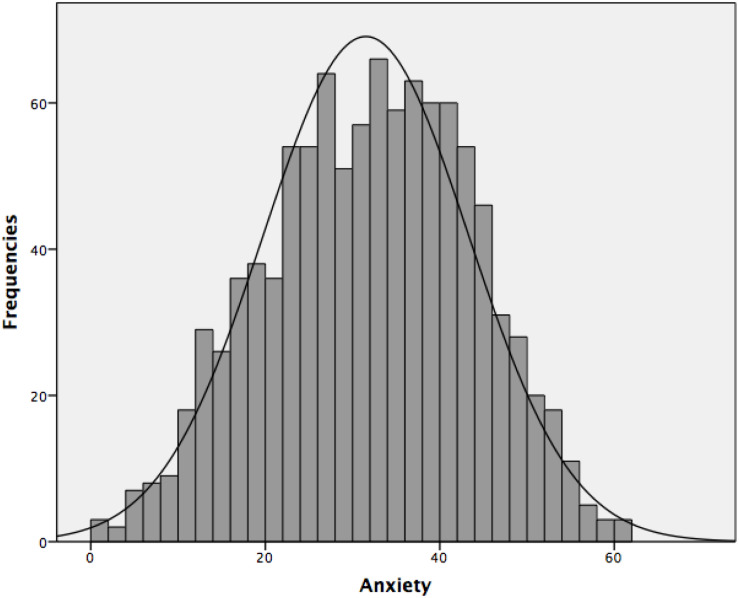
Histogram of the distribution of anxiety in the general sample.

**FIGURE 2:**
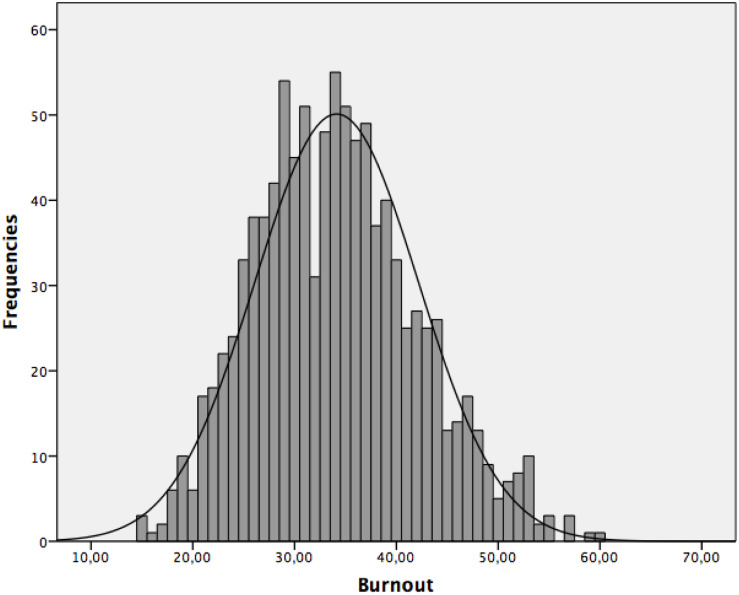
Histogram of the distribution of burnout in the general sample.

Our first objective aims to descriptively analyze the levels of anxiety and burnout in the complete sample participants. Regarding anxiety, the distribution as shown in Figure 1 is distributed slightly above the possible medium value (30), which indicates that our participants had a medium-high level [(Mean = 31.53 (SD = 11.77)]. Regarding burnout [(Mean = 34.13 (SD = 8.04)], the distribution was grouped slightly below the central point, as can be seen in Figure 2.

For our second objective, we explored the association between anxiety and burnout in students who were taking the university access assessment test (post-lockdown situation). For this purpose, we only worked with this sub-group of the total sample. A Pearson bivariate correlation test was carried out (*r* = 0.46, *p* = 0.00), which clearly indicated that participants’ higher levels of anxiety were significantly associated with higher levels of burnout.

**FIGURE 3:**
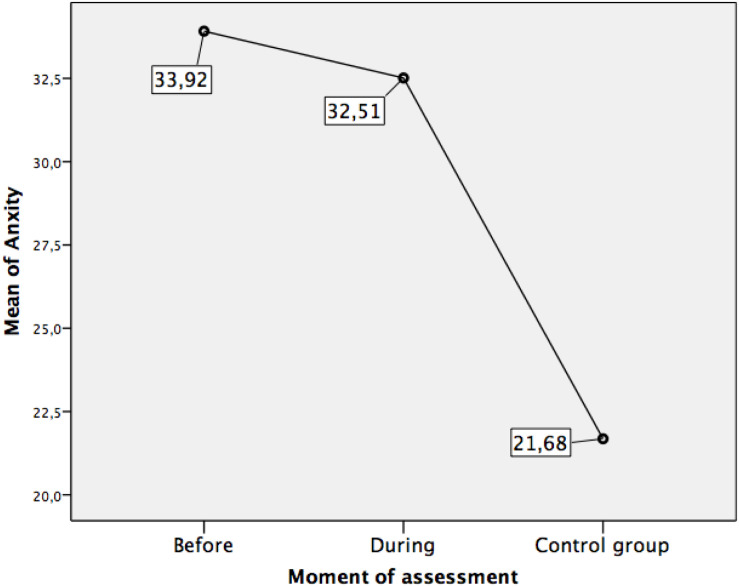
Means in anxiety according to the assessment moment.

**FIGURE 4:**
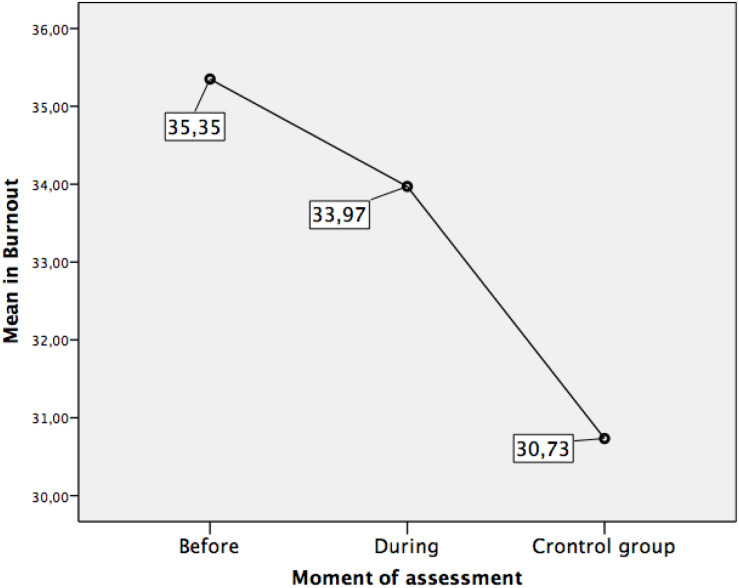
Means in burnout according to assessment moment.

Our third objective was to determine possible differences in anxiety and burnout between the three established conditions: (a) at least 2 month before the exams, when the students were confined; (b) on the days of the exam (just after lockdown, still with restrictive measures applied by the health authorities); and (c) first-year university students who had taken the exam in the past in normal social circumstances (control group). Therefore, we worked with the complete sample of study participants and carried out the respective mean comparisons (univariate ANOVA) with an additional minimal significant difference (MSD) contrast. The ANOVA indicated significant differences in anxiety between the three evaluation conditions, *F*(2,1016) = 74.45, *p* = 0.000. The same relationship was also observed in burnout, *F*(2,1007) = 20.03, *p* = 0.000. The specific mean differences in burnout and anxiety between the three groups of participants are detailed in Table 2 and Figures 3, 4.

**TABLE 2:** MSD comparison of academic burnout and anxiety according to the moment of assessment.

**(I) Assessment moment**	**(J) Assessment moment**	**Mean differences (I,J)**	**Sig.**
Anxiety			
(a) Assessment at least 2 month before the examinations (lockdown situation). (Mean = 33.92; SD = 10.84).	(b) Assessment during the evaluation test (post-lockdown situation). (Mean = 32.51; SD = 11.70).	1.41	0.06
(a) Assessment at least 2 month before the examinations (lockdown situation). (Mean = 33.92; SD = 10.84).	(c) Assessment before the COVID-19 pandemic, in first-year students (social normality situation – Control group). (Mean = 21.68; SD = 9.57).	12.24	0.00
(b) Assessment during the evaluation test (post-lockdown situation). (Mean = 32.51; SD = 11.70).	(c) Assessment before the COVID-19 pandemic, in first-year students (social normality situation – Control group). (Mean = 21.68; SD = 9.57).	10.83	0.00

Burnout			
(a) Assessment at least 2 month before the examinations (lockdown situation). (Mean = 35.35; SD = 7.52).	(b) Assessment during the evaluation test (post-lockdown situation). (Mean = 33.97; SD = 8.54).	1.38	0.01
(a) Assessment at least 2 month before the examinations (lockdown situation). (Mean = 35.35; SD = 7.52).	(c) Assessment before the COVID-19 pandemic, in first-year students (social normality situation – Control group). (Mean = 30.73; SD = 7.34).	4.62	0.00
(b) Assessment during the evaluation test (post-lockdown situation). (Mean = 33.97; SD = 8.54).	(c) Assessment before the COVID-19 pandemic, in first-year students (social normality situation – Control group). (Mean = 30.73; SD = 7.34).	3.24	0.00

Concerning anxiety, the differences were generally significant but not exactly between the three groups. The highest levels of anxiety were observed in the group evaluated at least 2 month before the access exam (whose members were in a confinement situation), but the differences between this group and the group evaluated while taking the exam were not significant. The control or normal-functional group, assessed when already enrolled in the university, presented significantly lower—almost minimal—anxiety levels.

Concerning burnout, the differences between the three groups were significant. The confined participants evaluated at least 2 month before the university access exam had higher levels than the students who were actually taking the exam, and both groups had higher levels than the control group.

**FIGURE 5:**
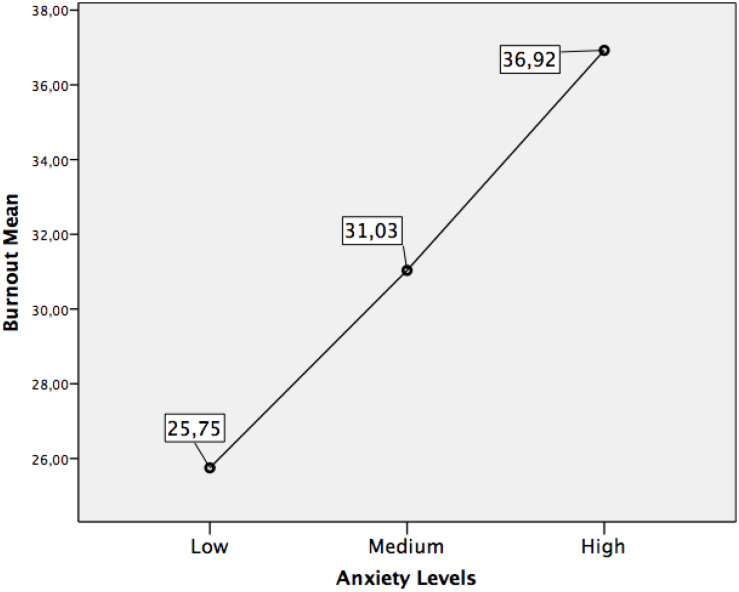
Means in burnout according to anxiety level.

Finally, the fourth objective analyzed possible differences in burnout according to different levels of anxiety in the students who took the university access exam. For this purpose, we only used the subsample of post-lockdown students who were taking the access exam at that moment, through a univariate ANOVA with an additional MSD contrast. The results indicated significant differences in burnout as a function of different levels of anxiety, *F*(2,377) = 38.77, *p* = 0.000]. The levels of anxiety were established following the scaling criteria proposed by the authors of this inventory (Spielberger et al., 2002), thus proceeding to the extraction of three groups of students according to their level in this variable. The difference in mean burnout between these three groups of participants is detailed in Table 3 and the descriptive results are illustrated in Figure 5.

**TABLE 3:** MSD comparison test in academic burnout according to anxiety levels.

**(I) Anxiety level**	**(J) Anxiety level**	**Mean differences (I,J)**	**Sig.**
Low (Mean = 25.75; SD = 6.43).	Medium (Mean = 31.03; SD = 7.62).	5.28	0.00
Low (Mean = 25.75; SD = 6.43).	High (Mean = 36.92; SD = 8.04).	11.17	0.00
Medium (Mean = 31.03; SD = 7.62).	High (Mean = 36.92; SD = 8.04).	5.89	0.00

As can be seen in Figure 5, burnout levels were higher as anxiety levels rose. All comparisons between the means of these three groups of participants were significant.

## Discussion and Conclusion

As mentioned, to date, there have been few studies about the presence of anxiety and burnout and the relationship between them in students facing the university access exam in Spain. Our study also analyses the presence of anxiety and burnout during lockdown due to the COVID-19 pandemic and in the so-called “new normality” (post-lockdown situation) in Spain.

That is why we first proposed to determine the presence of these variables in a sample of students. Our results have shown relatively high levels of anxiety and somewhat less burnout. These results agree with those found by other studies with different samples, where test anxiety is considered habitual and high (Fernández-Castillo, 2013; von der Embse et al., 2018; Fernandez-Castillo and Caurcel, 2019).

For our second objective, to determine the association between anxiety and burnout, in this case, we only analyzed the sample of students who were taking the university access exam. The significant and positive association between anxiety and burnout had been observed in students taking exams, but not in the case of the university access exam in Spain, in a national post-lockdown situation or with exceptional social interaction measures. However, these results are consistent with previous studies carried out in other circumstances (Chust-Hernández et al., 2019).

The results of our third objective were also highly interesting. We wanted to explore possible differences in anxiety and burnout between the three assessment moments or conditions: at least 2 month before the exam (lockdown), while taking the exam post-lockdown, and in students already admitted to the university (normality). The comparisons of means revealed significant differences only between the lockdown condition (months before the examination) and the control group as well as between the post-lockdown condition (on the examination days) and the control group. However, no significant differences were found between the lockdown and post-lockdown situations, indicating that the levels of anxiety were already high at least 2 month before the access exam (when students were in lockdown), and remained so until the days of the exam. Anxiety levels were significantly lower in students who were enrolled in the university. This result coincides with others that had already found very high levels of anxiety in this type of exam in other countries (Yildirim, 2007; Kavakci et al., 2014; Unalan et al., 2017). The result indicates that the exposure to anxiety is probably maintained for months (probably throughout the entire last high school course, which focuses on preparing students for the university access exam) and it possibly increased due to the lockdown when these students could not attend their educational centers in person and had to prepare the exam at home. This also indicates these students’ high concern, especially due to anticipation, which may also have consequences for their psychological health. Taking these results into account, the study of the possible long-term psychological and behavioural consequences should be addressed in future studies.

The comparative analysis of the burnout means in the three groups of students also revealed significantly different levels of emotional and physical fatigue regarding their academic work. It is noteworthy that at least 2 month before the access exam (during the lockdown), participants showed an even higher level of burnout than during the days the exam was held. This result could be explained because, in the days of the exam, the end of a process that had lasted practically the entire academic year is perceived, which is liberating. As expected, the group with the lowest burnout level was the group of university students. This control group contextualizes the result of anxiety in the other two groups that face the access exam.

Finally, to delve into the relationship between anxiety and burnout, our fourth objective explored possible differences in burnout according to different levels of anxiety only in the group of students who were taking the access exam. These students faced the access exam in exceptional historical circumstances, having been confined for 2 month before the exam and surrounded by social-health measures that were also unique (including the use of a face mask, maintaining social distance, etc.). The differences in burnout between the low, medium, and high anxiety groups were significant. The relationship between higher anxiety and higher levels of burnout is clear and significant and in line with the findings of other studies in other populations (Chust-Hernández et al., 2019). These results more solidly link the relationship between these two variables during university access exam, suggesting the need to address anxiety to reduce burnout levels in these students.

To conclude, our study shows that coping with university access exams, considered high-stakes tests, generate high levels of anxiety and concern in these students, an aspect that was already studied in other samples and countries (Segool et al., 2013). Anxiety seems more intense during the lockdown due to the COVID-19 pandemic and also afterward, in the post-lockdown situation. Our results also verified that anxiety is maintained for very long periods of time, at least 2 month before the exam. Future studies could explore anxiety for even longer intervals, as well as the consequences of exposure to these circumstances. It may also be of interest to explore other aspects that may be present in this context, such as other emotional disturbances or negative effects that these exams may have on students. It is already known that greater perceived importance of an exam could be associated with a higher perception of threat and concern in those who face it. Our study is pioneer in detecting and relating these variables in these students and these academic circumstances, not only the exceptional situation derived from the COVID-19 pandemic but also the circumstance that most students who want to access the university in Spain must undergo.

A possible limitation of our study refers to the sample of participants used. Specifically, given the type of sampling carried out, the sample may not represent the reference population and therefore our results must be taken with caution in order to generalize. Despite it, our results also open a line of reflection on these aspects of our educational system, which could harm students’ health. It should be kept in mind that anxiety is one of the most frequent psychological disorders worldwide (World Health Organization, 2017), and is associated with a multitude of other problems. The reduction of anxiety and burnout should be a concern, both for educators and policy-makers in this educational and evaluation area.

## Data Availability Statement

The raw data supporting the conclusions of this article will be made available by the authors, without undue reservation.

## Ethics Statement

Ethical review and approval was not required for the study on human participants in accordance with the local legislation and institutional requirements. Written informed consent to participate in this study was provided by the participants’ legal guardian/next of kin.

## Author Contributions

AF-C conceived and designed the study, obtained the information, performed the statistical analysis, interpretation of the results, and wrote the manuscript.

## Conflict of Interest

The author declares that the research was conducted in the absence of any commercial or financial relationships that could be construed as a potential conflict of interest.
